# Impact of cement type and abutment height on pull-off force of zirconia reinforced lithium silicate crowns on titanium implant stock abutments: an in vitro study

**DOI:** 10.1186/s12903-021-01958-6

**Published:** 2021-11-19

**Authors:** Lisa Müller, Angelika Rauch, Daniel R. Reissmann, Oliver Schierz

**Affiliations:** 1 Nossen, Germany; 2 Department of Prosthodontics and Materials Science, University of Leipzig, Liebigstrasse 12, 04107 Leipzig, Germany; 3 Department of Prosthetic Dentistry, University Medical Center Hamburg-Eppendorf, Martinistrasse 52, 20246 Hamburg, Germany

**Keywords:** Ceramics, Fixed dental prosthesis, Implant cement, Prosthetic dentistry, Provisional cement, Semi-permanent cement, Abutment height, Zirconia reinforced lithium silicate (ZLS)

## Abstract

**Background:**

Pull-off forces of cement-retained zirconia reinforced lithium silicate (ZLS) in implant-supported single crowns on stock titanium abutments with respect to abutment height and implant cement were evaluated and compared.

**Methods:**

Pull-off force of ZLS crowns on stock titanium abutments was evaluated concerning dental cement and abutment height. A total sample size of 64 stock abutments with heights of 3 mm (n = 32) and 5 mm (n = 32) was used. The ZLS crowns were cemented with four different types of cement (one temporary, two semi-permanent, and one permanent). After cementation, water storage, and thermocycling each sample was subjected to a pull-off test using a universal testing machine.

**Results:**

The temporary cement showed the least pull-off force regardless of abutment height (3/5 mm: means 6 N/23 N), followed by the semi-permanent methacrylate-infiltrated zinc oxide cement (28 N/55 N), the semi-permanent methacrylate-based cement (103 N/163 N), and the permanent resin composite cement (238 N/820 N). Results of all types of cement differed statistically significantly from each other (*p* ≤ .012). The type of implant cement has an impact on the pull-off force of ZLS crowns and titanium abutments.

**Conclusions:**

Permanent cements present higher retention than semi-permanent ones, and temporary cements present the lowest values. The abutment height had a subordinate impact.

## Background

In everyday clinical practice, implants have become a beneficial treatment, especially to replace single teeth and to restore function, esthetics, and patient satisfaction with the oral appearance. Due to modern developments in interdisciplinary research on dental materials, implant prosthetics, and surgery, and more as well as increasing survival rates, the choice and popularity of dental implants among dentists and patients has increased [1]. However, debates about favored materials and techniques in implant prosthetics continue until today [2, 3].

The retrievability of implant-supported single crowns (iSC) and multi-unit fixed dental prostheses (iFDPs) is substantial for the maintenance of implants, complication management, and replacement of the prosthesis [4]. In general, restorations can be screw-retained, cement-retained, or fixed with a combination of both [5]. Regarding the survival of the implant, no clinically relevant differences were described between the rates of cement- or screw-retained iSCs during the first five years. Thus, both fixation methods can be recommended for implant-supported restorations [3]. In the presence of limited interocclusal space of 4 mm, screw-retained structures are preferred [6]. Advantages of cement-retained iSCs are the provision of a more passive fit of the crown, as well as better esthetical and occlusal features [7]. Due to reduced costs and the simplicity of the procedure, 60% of German clinical practitioners prefer cementation on implant abutments [5, 8]. In comparison to screw-retained crowns conventional cementation can avoid common technical complications such as screw loosening or mechanical damage of the implant components. Furthermore, no special devices are needed for the conventional approach and the procedures follow the same routine as for natural abutment teeth [7].

However, for the long-term success of dental prosthesis implant-related biological complications are important too. Biological complications refer to soft and hard tissue diseases. One of the main disadvantages of cemented crowns is the risk of undetected cement residue that could lead to inflammatory reactions and peri-implant complications, like peri-implantitis, peri-implant mucositis, soft tissue hypertrophy, or recession up to bone loss and necessary deplantation [9]. Studies prove, that biological challenges are higher with cement-retention than screw-retained implant-supported fixed dentures [10, 11].

Dental cements can be classified as temporary, semi-permanent, and permanent. Temporary cements have been developed for short-term fixation and intended debonding. Therefore, they are inherently weak and soluble in the oral environment [12]. For the cementation of iSCs and iFDPs, a class of semi-permanent cements has been developed that accomplishes a chemical bond between the abutment and the restoration [13, 14]. These cements should be characterized by sufficient strength, which avoids unintentional decementation and provides reliable intended decementation when needed. That aspect showed to be tremendous progress in the development of dental cements [15–17]. Many authors and clinicians recommend temporary cementation to facilitate retrievability of iSCs and iFDPs without damage [18, 19].

Factors affecting cemented iSCs and iFDPs are similar to those on natural teeth and are characterized by type of luting agent, film thickness, the roughness of the bonding surface, taper, width, and height of the abutments [13, 15, 20]. In situations of limited interocclusal space, shorter abutments are mandatory, even if the small height may become a limiting factor for clinical success. Therefore, an investigation on pull-off forces regarding different abutment heights and different types of implant cements can provide valuable information for clinicians.

The purpose of this study was to evaluate and compare pull-off force of cement-retained zirconia reinforced lithium silicate iSCs on stock titanium abutments with respect to abutment height and implant cement. Regarding the pull-off force, the null-hypothesis states that there is no significant difference in iSCs made from ZLS bonded with four different dental implant cements to stock titanium abutments. The secondary hypothesis states that there is no difference in pull-off force regarding the abutment height.

## Methods

For sample size calculation, data from preliminary pull-off tests were used. A pull-off force difference of 50 N between both heights was assumed as clinically relevant. Estimating a standard deviation of 30 N and a power of 0.9, calculations revealed a sample size of 8 per group (in total 64 samples when using four different cements and two different abutment heights) using a two-sample test (STATA 15.1 College Station, TX: StataCorp LLC).

A number of 24 stock titanium abutments (CAMLOG iSy® Esthomic® abutments, CAMLOG Vertriebs GmbH, Germany) were physically available for the testing process. The abutments were delivered with a pronounced chamfer and tapering of 7.5 degrees. Half of the abutments were customized manually to a height of 3 mm and the other was customized to a height of 5 mm by using a Red Milling Cutter (A. M. Edelingh M + W Dental, Germany). A mold was designed and fabricated, with the help of which the implant analogs were embedded in polymer blocks (Paladur®, Kulzer GmbH, Germany). Each stock abutment was screw-retained to the implant analog with 20 Ncm torque with a manufacturer-supplied manual torque controller (CAMLOG Biotechnologies AG, Germany). After cleaning with isopropanol, screw access openings were filled with a temporary resin composite material (Telio CS, Ivoclar Vivadent AG, Liechtenstein). A gingival mask was handmade with addition-curing vulcanizing silicone (GumQuick, Dreve Dentamid GmbH, Germany) for digitization of the stock abutments (Omnicam, CEREC Premium SW 4.5, Dentsply Sirona Deutschland GmbH, Germany).

All shorted stock abutments were scanned and an associated individual iSC was designed as upper left second premolars using a form template (inLab SW 4.6.1, Dentsply Sirona Deutschland GmbH, Germany). A spacer of 120 μm was set [21]. A number of 24 monolithic ceramic crowns made of zirconia reinforced lithium silicate (VITA SUPRINITY®, VITA Zahnfabrik H. Rauter GmbH & Co. KG, Germany) were milled (inLab MCXL, Dentsply Sirona Deutschland GmbH, Germany) from ZLS blocks of 12 × 14 × 18 mm.

**Fig. 1:**
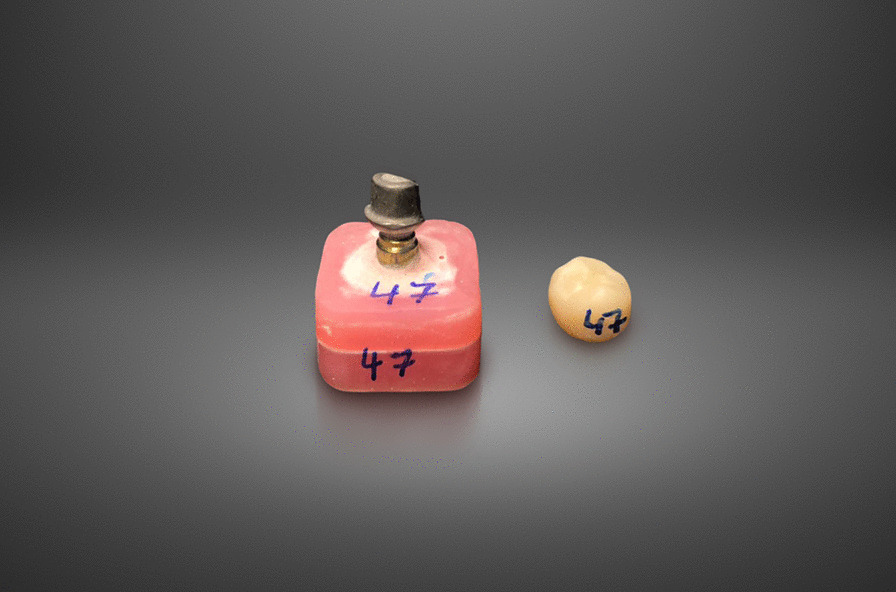
Exemplary sample; implants analogue embedded in polymer blocks and milled and crystalized monolithic crown

The milled crowns were separated from the block and the lug was removed with a diamond-coated milling instrument. The iSCs were manually cleaned, air-dried, and crystallized according to the manufactures’ instructions (Programat EP5000; Ivoclar Vivadent AG, USA). All crowns were polished, as recommended by the manufacturer. To reassociate the iSC to its abutment, each sample was numbered (Fig. 1). The bonding surfaces of the finished crowns were airborne abraded with 50 μm Al_2_O_3_-particles at a pressure of one bar, duration of 10 s, and a distance of 10 mm [22]. All restorations and abutments were purged with isopropanol (70%) and air-dried immediately before cementation.

The cements (Table 1) were applied into the inner lumen of the restoration up to the cervical third of the crown and carefully placed on the corresponding stock abutment. Before the cementation with permanent resin composite cement, the inner surface of the crown was conditioned for 60 s (Monobond Plus, Ivoclar Vivadent GmbH, Germany) and the surplus removed by air current. Subsequently, all restorations were placed on the stock abutment and kept in place for 10 s. Thereafter, all samples were axially loaded with 50 N for about 10 min under room conditions (21 °C and 50% humidity).

**Table 1:** Classification and main components of the used luting agents

Trading name (abbreviation)	Type	Manufacturer	Classification	Main components
TempBond NE® (TBNE)	Zinc oxide cement (chemical curing)	KerrHawe SA, Swizerland	Temporary [17]	Zinc oxide, zinc acetate dihydrate
Harvard Implant® (HI)	Methylacrylate-infiltrated zinc oxide cement (dual curing)	Havard Dental Intl GmbH, Germany	Semi-permanent	Zinc oxide, multifunctional methacrylates
Premier® Implant Cement™ (PI)	Methacrylate-based cement (chemical curing)	Premier® Dental Products Company, Pennsylvania USA	Semi-permanent [17]	Triethylenglycoldi-methacrylate, fumed silica, 2-hydroxy-ethylmethacrylate, benzoyl peroxide, resin
SpeedCEM® Plus (SCP)	Composite cement (self-adhesive, dual curing)	Ivoclar Vivadent GmbH, Germany	Permanent	Triethylenglycoldi-methacrylate, polyethylenglycoldi-methacrylate, ytterbium trifluoride, 10-methacryloyl-oxydecyldihydrogen-phosphat

After 24 h water storage at 37 °C, all samples were loaded with 37,500 thermal cycles (Thermocycler Haake DC 10, W 15, Thermo Haake GmbH, Germany), corresponding to a lifetime of approximately 4 years in vivo [23], making the study comparable to clinical short-term studies [5]. For thermocycling, two water baths were filled with 5 °C and 55 °C tempered, distilled water with a dwell time of 30 s and a transition time of 10 s. Both temperature data correspond to the median value of the measured maximum and minimum temperatures in the oral cavity [23].

**Fig. 2:**
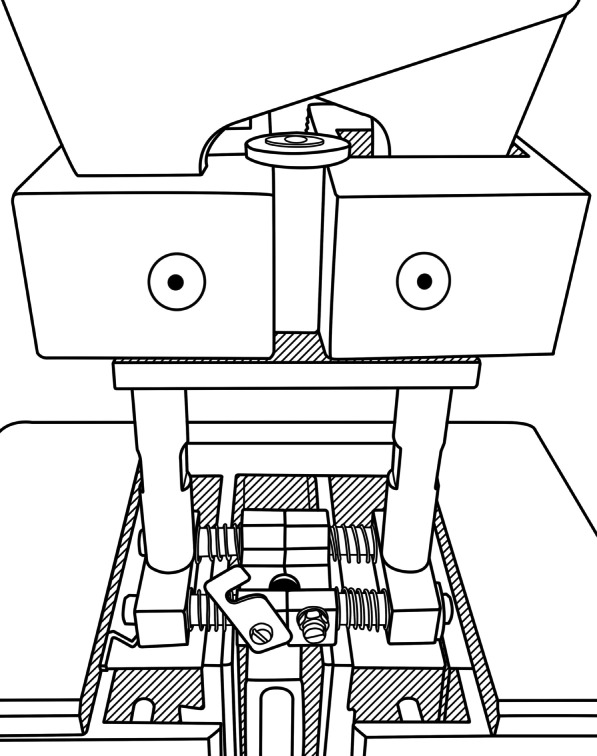
Custom-made device setting in the universal testing machine, for measurement of the pull-off force

Afterwards, each sample was clamped into a custom-made device setting (Fig. 2) (Zwick Roell Z010, Test X pert® II V2.2, ZwickRoell GmbH & Co. KG, Germany).

The crowns were axially removed with a crosshead speed of 2 mm/min and a preload of 4 N. The Adhesive Remnant Index [24] was registered according to Table 2 immediately after the crown removal, to record the quantitative affinity of the cements for abutment and crown surfaces.

**Table 2:** Codes for visual, quantitative classification of the cement remnants on the abutments and in the crowns (modified Adhesive Remnant Index [24])

Codes	Interpretation
1	Cement adheres completely to the abutment
2	More than 50% of the cement adheres to the abutment
3	More than 50% of the cement adheres in the crown
4	Cement adheres completely in the crown

**Fig. 3:**
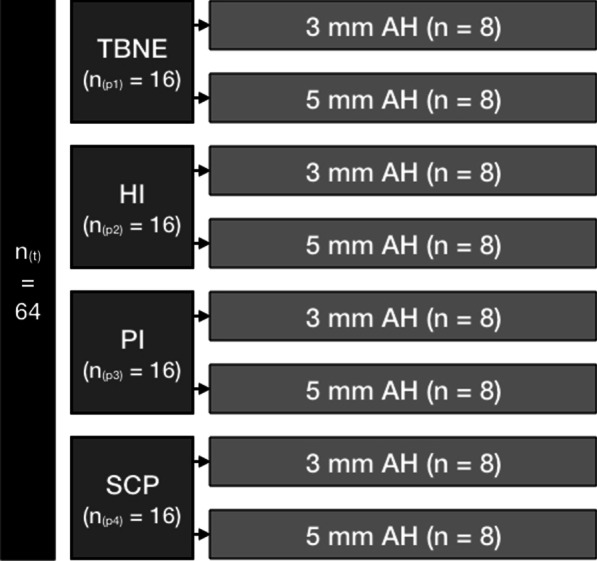
Flowchart; TBNE = TempBond NE® a temporary zinc oxide-based cement, HI = Harvard Implant® a semi-permanent methacrylate-infiltrated zinc oxide cement, PI = Premier® Implant Cement™ a semi-permanent methacrylate-based cement, SCP = SpeedCEM® Plus a permanent composite cement, AH = abutment height

The available 24 stock abutments and belonging crowns of both heights were refurbished up to two times, so a total amount of 64 tests in both abutment heights could be performed. The intactness and complete purification of the inner and outer surface of crown and abutment were visually inspected with magnifying glasses (magnification 2.5). Every luting agent was tested eight times with each abutment height. All 64 samples (Fig. 3) were processed in randomized order according to the flowchart.

Data were statistically analyzed with SPSS (V27.0.0.0, IBM Deutschland GmbH, Germany) applying one-way ANOVA to test whether there was a difference concerning the applied type of cement, and *t*-test to figure out differences regarding abutment height and the comparison between the 4 cements. To estimate the effect size, Cohen’s *d* was calculated and interpreted after Sawilowsky [25]. The results of the Adhesive Remnant Index were statistically evaluated by using the Kruskal–Wallis-Test, and Mann–Whitney-U-Test was used to figure out differences regarding the different cements.

## Results

The temporary zinc oxide-based cement (TBNE) showed the least mean pull-off force regardless of the abutment height (3 mm/5 mm: means 6 N/23 N), followed by the semi-permanent methacrylate-infiltrated zinc oxide cement (HI) (28 N/55 N), the semi-permanent methacrylate-based cement (PI) (103 N/163 N) and the permanent composite cement (SCP) (238 N/820 N). In the temporary cement group, more than half of the crowns debonded spontaneously. Samples bonded with the two strongest implant cements (PI and SCP) showed damages of the crowns while removal in both abutment heights (PI: 12.5%; SCP: 25.0%).

The statistical analysis revealed a significant difference and a huge effect size (*d* > 2.0) for abutment height when using SCP and HI. There were no statistically significant differences between the pull-off forces regarding the abutment height in the groups of PI and TBNE (Table 3). Moreover, statistically significant differences in pull-off forces between all cements and an abutment height of 3 mm and 5 mm were detected, as well as very large to huge effect sizes (Tables 4, 5). Solely the pairing of TBNE and HI on 5 mm abutment height presented no statistically significant differences in pull-off force (Table 5) and small effect size (*d* > 0.2).

**Table 3:** Pull-off force with respect to cement and abutment height

Cement	3 mm abutment height		5 mm abutment height		Statistical significance	Effect size
	Mean (SD) in N	Range in N	Mean (SD) in N	Range in N		
TBNE	6 (12)^6^	0–27	23 (21)^3^	0–54	*p* = 0.074	0.99
HI	28 (6)	22–37	55 (39)	8–102	*p* < 0.001*	0.97
PI	103 (40)^#1^	49–180	163 (96)^#1^	71–310	*p* = 0.132	0.83
SCP	238 (131)^#2^	66–525	820 (180)^#2^	550–1170	*p* < 0.001*	3.70

(a) Asterisks (*) mark statistically significant differences

(b) X^#n^ marks the count of crown fractures

(c) X^n^ marks the count of premature debonding

**Table 4:** Probability of statistically significant differences and effect size of cement pairings 3 mm abutment height

TBNE		2.4	3.3	2.5
HI	*p* = 0.001*		2.6	2.3
PI	*p* < 0.001*	*p* = 0.001*		1.4
SCP	*p* = 0.002*	*p* = 0.003*	*p* = 0.023*	
	TBNE	HI	PI	SCP

(a) Asterisk (*) marks statistically significant differences

(b) Interpretation effect size: > 0.2 small; > 0.5 medium; > 0.8 large; > 1.2 very large; > 2.0 huge

**Table 5:** Probability of statistically significant differences and effect size of cement pairings 5 mm abutment height

TBNE		0.3	2.0	6.2
HI	*p* = *0.068*		1.5	5.9
PI	*p* = 0.004*	*p* = 0.002*		4.6
SCP	*p* < 0.001*	*p* < 0.001*	*p* < 0.001*	
	TBNE	HI	PI	SCP

(a) Asterisk (*) marks statistically significant differences

(b) Interpretation effect size: > 0.2 small; > 0.5 medium; > 0.8 large; > 1.2 very large; > 2.0 huge

Table 6 depicts the residues of the remnant cement on an abutment and/or crown. The temporary cement left most of the residue on the titanium surface of the abutment. The semi-permanent and permanent cements quantitatively rather remained on the inside of the crown after removal from the abutment. The Kruskal–Wallis-Test shows statistically significant differences between all cements regarding their cement residues (*p* < 0.001).

**Table 6:** Observed cement remnants; for codes refer to Table 2

Cement	Code (modified adhesive remnant index)			
	Code 1 (%)	Code 2 (%)	Code 3 (%)	Code 4 (%)
TBNE	18.75	68.75	12.50	0
HI	6.25	6.25	6.25	81.25
PI	0	0	25	75
SCP	0	0	68.75	31.25

Solely, no statistically significant difference was found between PI and HI (Table 7).

**Table 7:** Probability of statistically significant differences regarding observed cement remnants

TBNE				
HI	*p* < 0.001*			
PI	*p* < 0.001*	*p* = 0.834		
SCP	*p* < 0.001*	*p* = 0.023*	*p* = 0.015*	
	TBNE	HI	PI	SCP

Asterisk (*) marks statistically significant differences

## Discussion

Both null hypotheses stating that there is no difference in pull-off force between the chosen types of cement as well as that there is no difference in pull-off force between the abutment heights has to be rejected. Permanent implant-cements presented a higher pull-off force than semi-permanent ones. However, the latter have a higher pull-off force than temporary cements. The unintended retention loss of 56.0% of the temporary cemented (TBNE) crowns was disproportionally high. With the semi-permanent methacrylate-based cement (PI), the second-highest results of pull-off forces could be achieved but crown fractures were present. The pull-off force observed in semi-permanent methacrylate-infiltrated zinc oxide cement (HI) was high enough to avoid unintentional losses and low enough to avoid crown fractures during the removal.

This study confirmed that permanent cements (SCP) show higher pull-off forces than semi-permanent ones (like PI and HI), which in return show higher pull-off forces than temporary cements (TBNE) [14]. As shown in previous studies, temporary cements presented increased unintentional decementations [5]. Glutekin et al. [17] compared seven implant-cements and observed a significant difference between semi-permanent methacrylate-based cement (PI) and TBNE, which is consistent with the results of this study. Lopez et al. [19] demonstrated that resin-based cements such as PI and HI have statistically significantly higher pull-off force compared to the temporary cement group and regardless of the crown material. Silva et al. [26] observed similar results by testing zirconia crowns on titanium abutments. Due to its low solubility and high mechanical and sealing properties, resin-based semi-permanent cements are most effective in preventing microleakages [27]. This might explain the comparable but not equal results of PI and HI whose pull-off forces were less than those of the permanent cement but higher than for the provisional cement.

According to the observed cement remnants, all cements differ statistically significant from each other, except the two semi-permanent cements. Semi-permanent and permanent cement groups showed a higher affinity for the ZLS surface of the crowns than for the titanium surface of the abutments. The high adhesion of the cement residues to the crown surface of the permanent cement may be due to the previous silanization [28, 29]. The highest pull-off forces were achieved by the permanent composite cement, and therefore the risk of damaging the restoration is increased, which is corroborated by the fractured crowns during the removal trial.

The null hypothesis that the abutment height has no relevant impact on the pull-off force has to be partially rejected. Except for the use of SCP and HI the abutment height showed no statistically significant impact on the pull-off force. Other studies showed an increasing pull-off force by increasing abutment height (from 4 to 6 mm) in permanent cements, which corroborates the result of this investigation [30]. Pull-off forces of temporary and semi-permanent cements luting ZLS crowns to titanium abutments were not relevantly affected by different abutment heights in other studies [26]. Sarfaraz et al. [15] used PI and TBNE and presented comparable results examining height differences of 1.5 mm. The abutment height has tremendous importance in everyday clinical practice and is supported by the large effect sizes in our study. Customized abutments are an existing alternative that shows a lower risk of crown loosening in comparison to stock abutments [31]. In cases of limited space customized abutments should be considered to improve retention. The combination of different surface treatment combinations of sandblasting and primer application could improve retrievability when using stock abutments [32]. Other studies observed significant differences in pull-off forces affected by abutment height when using titanium abutments and zirconia crowns or titanium abutments and cobalt-chromium crowns [33, 34].

In this study, high standard deviations were found. This phenomenon may be partially attributed to the reuse of the crowns and stock titanium abutments, which were reset manually by sandblasting and cleansing. Potentially repeated refurbishing of the stock abutment and crown surface may cause alteration in surface roughness of the titanium and ZLS surface [35, 36]. For this reason we limited the reuse of the crowns to two times. Naumova et al*.* [37] proved that a combination of sandblasting and repeated cementation of implant-supported cobalt-chromium crowns leads to reduced retention force independent of the luting agent. Additionally, the adjustment of the abutment heights and the closing of the screw access hole were carried out manually. In summary, it could be considered that the surfaces of each stock titanium abutment were slightly different in each test. Nonetheless, the procedure reflects a standard situation in daily dental practice.

## Conclusions

For clinical purposes, it seems impossible to define the “best” cement for all implant situations. Instead, a ranking of types of cement should be sought and dependence of the abutment height should be kept in mind [21]. Due to the high number of spontaneous decementations and low pull-off forces, the use of a temporary cement for luting cannot be recommended. In cases of retention loss, clinicians can choose alternative luting agents in ascending order [13, 16]. Based on the results made in the present investigation a ranking of implant cements can be made as follows: TBNE < HI < PI < SCP. However, semi-permanent cementation, here with PI and HI, seems to be the most suitable in the case of luting ZLS crowns to stock titanium abutments, as the pull-off forces were high enough to ensure a low risk of unintended debonding and low enough for a predictable successful attempt of intended debonding. For further investigations, it would be a necessity to define the separation between temporary, semi-permanent, and permanent types of cement. Within our investigation and the limitations of this in-vitro study, the semi-permanent methacrylate-infiltrated zinc oxide cement (HI) presented as favorable for semi-permanent cementation of ZLS crowns on titanium abutments.

## Data Availability

The datasets analyzed during the current study are available from the corresponding author on reasonable request.

## Abbreviations

ZLS: Zirconia reinforced lithium silicate
iSC: Implant-supported single crowns
iFDPs: Multi-unit fixed dental prostheses
TBNE: TempBond NE®
HI: Harvard Implant®
PI: Premier® Implant Cement™
SCP: SpeedCEM® Plus
